# NMR contributions to structural dynamics studies of intrinsically disordered proteins^☆^

**DOI:** 10.1016/j.jmr.2013.11.011

**Published:** 2014-04

**Authors:** Robert Konrat

**Affiliations:** Department of Structural and Computational Biology, Max F. Perutz Laboratories, University of Vienna, Campus Vienna Biocenter 5, A-1030 Vienna, Austria

**Keywords:** Intrinsically disordered proteins, Protein meta-structure, Structural biology, Biomolecular NMR, EPR spectroscopy, NMR spin relaxation

## Abstract

Intrinsically disordered proteins (IDPs) are characterized by substantial conformational plasticity. Given their inherent structural flexibility X-ray crystallography is not applicable to study these proteins. In contrast, NMR spectroscopy offers unique opportunities for structural and dynamic studies of IDPs. The past two decades have witnessed significant development of NMR spectroscopy that couples advances in spin physics and chemistry with a broad range of applications. This article will summarize key advances in basic physical-chemistry and NMR methodology, outline their limitations and envision future R&D directions.

## Highlights

• Cross-correlated NMR spin relaxation probes motional coupling in IDPs.
• NMR and EPR provide insight into the conformational ensemble of IDPs.
• Domain elongation as valuable strategy for decoupling of motional modes in IDPs.
• Conformational substates of IDPs comprise basic protein structural motifs.

## Graphical abstract

**Figure:**
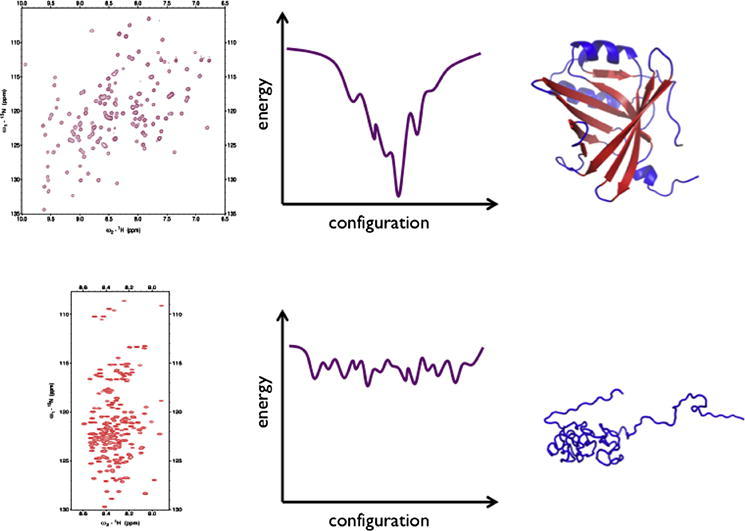

## 1 Introduction

Intrinsically disordered proteins (IDPs) have attracted a lot of attention in recent years based on the discovery of their importance in eukaryotic life and their central role in protein interaction networks. In contrast to their stably folded counterparts, IDPs feature a rather flexible nature. The efficient sampling of a vast and heterogeneous conformational space endows them with enormous potential to interact with and control multiple binding partners at the same time and it was thus proposed that this structural plasticity and adaptability allows IDPs to efficiently engage in weak regulatory networks (such as transcription regulation). The inherent structural flexibility of IDPs mandates the use of new experimental methods since X-ray crystallography, which is by far the most utilized tool in structural biology, cannot access these proteins in the completeness of their native states. NMR spectroscopy has been developed into a powerful structural biology technique complementing protein X-ray crystallography. In particular, it offers unique opportunities for structural and dynamic studies of IDPs. A fundamental problem in the structural characterization of IDPs is the definition of the conformational ensemble sampled by the polypeptide chain in solution. Often the interpretation relies on the concept of ‘residual structure’ where the observation of structural preferences and deviations from an idealized random coil devoid of any structural propensity are interpreted as prevalence of residual structures.

## 2 Experimental techniques

Over the last decade an NMR based methodological framework has emerged to characterize the structural dynamics of IDPs. Hydrogen exchange rates, NMR chemical shifts and residual dipolar couplings (RDC) can be used to evaluate local transient secondary structure elements with atomic resolution, whereas paramagnetic relaxation enhancement (PRE) reports on transient long-range contacts [1].

### 2.1 NMR spectral assignment of IDPs

**Fig. 1:**
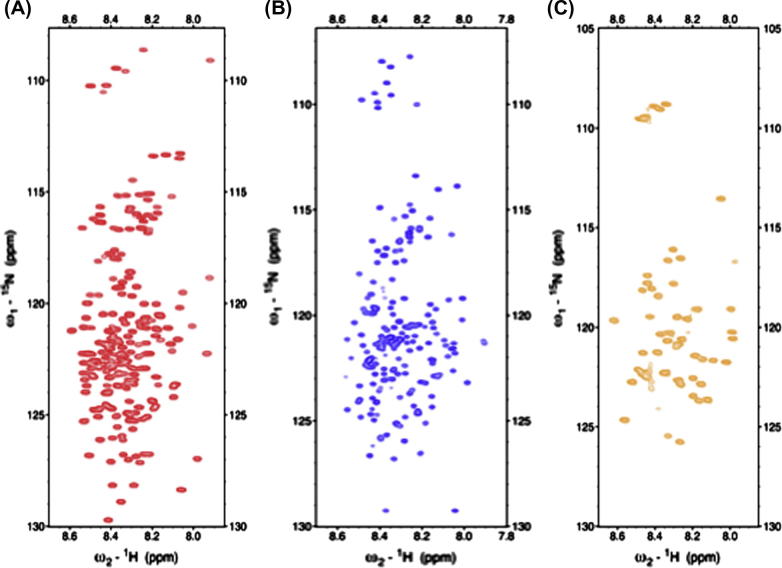
NMR spectral overlap in intrinsically disordered proteins. ^1^H^N^–^15^N HSQC spectra of IDPs are characterized by narrow spectral ranges and thus complicating sequential signal assignment. Spectra for the following IDPs are shown: (A) BASP1; (B) Osteopontin and (C) Tcf4.

**Fig. 2:**
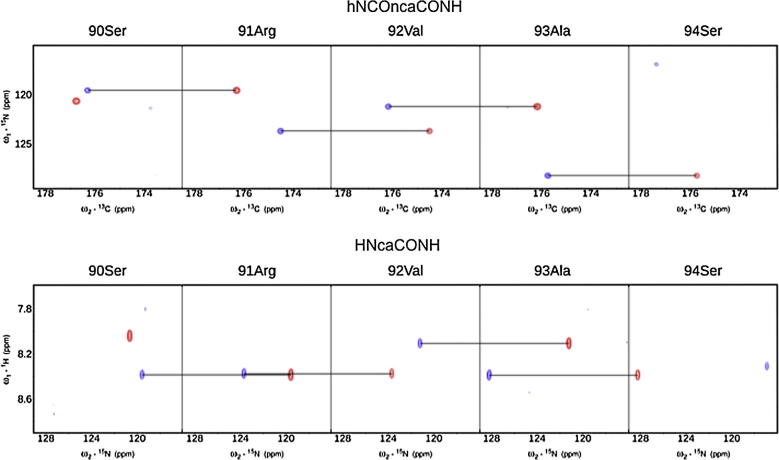
2D spectral planes for consecutive amino acids in MAP1B obtained by SMFT processing of the 5D randomly sampled signal. 2D cross-sections of (top) 5D hNCOncaCONH (N*_i_*–CO*_i_*_−1_ and N*_i_*_−1_–CO*_i_*_−2_) and (bottom) 5D HNcaCONH (HN*_i_*–N*_i_* and HN*_i_*_+1_–N*_i_*_+1_). Spins that were used for coherence transfer are depicted in lower case, while spins that are recorded during the indirect dimensions are given in upper case.

NMR signal assignment is well established for globular proteins. Typically, a suite of triple-resonance experiments is used to find sequential connectivities between neighboring residues. These experimental strategies rely on coherence transfer steps involving backbone ^13^C, ^15^N and ^1^H nuclei. Applications of these efficient techniques to IDPs are hampered because of severe spectral overlap and due to significant chemical exchange with bulk water that reduces ^1^H^N^ signal intensities leading to low signal-to-noise (S/N) ratios. Fig. 1 shows prototypical ^15^N–^1^H HSQC spectra obtained for different IDPs. While the latter can be partly overcome by measurements at low temperature and/or low pH, signal overlap problems required the development of novel NMR techniques. Capitalizing on improved instrumental sensitivities substantial improvements have been achieved due to: (i) non-uniform sampling technologies allowing for high-dimensionality experiments [2], (ii) faster acquisition of NMR experiments exploiting longitudinal relaxation enhancements [3] and (iii) direct detection of heteronuclei (^13^C) using cryoprobe technology and thereby circumventing problems of exchange broadening [4–6]. The signal assignment experiments overcome developed problems of poor dispersion and extensive signal overlap by utilizing non-uniform sampling of indirectly detected dimensions in combination with Sparse Multidimensional Fourier Transform (SMFT) processing. This enables the acquisition of high-resolution and high-dimensional spectra [2,7–9]. The particular advantage of these techniques is the fact that it is possible to calculate the Fourier integral for arbitrarily chosen frequency coordinates and thereby focusing only on those parts of the spectrum that contain actual peak information. The relevant regions can easily be identified based on some a priori knowledge of peak locations known from lower dimensionality spectra (2D, 3D) acquired before. Thus, frequency coordinates in these dimensions can be set to the exact peak frequencies extracted before and only low-dimensional cross-sections of the high-dimensional spectrum are calculated. Representative strip plots illustrating experimentally observed connectivities used for sequential signal assignment in IDPs are shown in Fig. 2.

Since NMR spectroscopy of IDPs (due to their favorable relaxation properties) is typically not limited by sensitivity but rather spectral resolution, relaxation-optimized detection schemes lead to further improvements. Recently, for example, a 3D BEST–TROSY-HNCO experiment has been described following this approach [10]. Additionally, given the fact that proline residues are highly abundant in IDPs, BT-optimized Pro-edited 2D ^1^H–^15^N experiments have been developed, that either detect ^1^H–^15^N correlations of residues following a proline (Pro-HNcocan) or preceding a proline (Pro-iHNcan) [10].

Given the availability of this powerful and robust NMR methodology spectral assignment of complex IDPs has been almost become a routine task and it can thus be anticipated that even larger and more complex IDPs will be amenable to this suite of NMR experiments.

### 2.2 NMR chemical shifts

Chemical shifts are known to be exquisite reporters of backbone conformation and therefore considerable efforts have been made to exploit this information to probe local structural propensities of IDPs (reviewed in [11]). In these applications deviations from random coil values are used to describe local geometries in IDPs and quantify local secondary structure elements (secondary structure propensities) have been proposed to describe local geometries in IDPs [12–14]. More sophisticated analysis scheme of NMR chemical shift data employ ensemble approaches developed by the groups of Forman-Kay [15], Stultz [16,17] and Blackledge [18]. Numerous applications have already been published and have been summarized in comprehensive reviews [11,19].

The application of NMR chemical shifts is not limited to structural analysis of proteins but they have also been shown to encode information about protein dynamics [20]. The inverse weighted sum of backbone secondary chemical shifts for Cα, CO, Cβ, N and Hα nuclei defines a so-called Random Coil Index (RCI). Although originally defined for the analysis of globular proteins, applications to IDPs will be feasible given the growing number of experimental studies.

### 2.3 Residual dipolar couplings (RDCs)

Dissolving proteins in anisotropic media leads to restricted overall reorientation, thus dipolar coupling interactions no longer average to zero leading to residual dipolar couplings (RDCs) that are experimentally observable in NMR spectra [21]. In IDPs, dynamic averaging of conformations differing in size and shape gives rise to non-zero RDCs. For example, negative ^1^D_NH_ RDCs are found for segments in which the NH vector is largely oriented perpendicular to the polypeptide chain (extended conformations). Conversely, positive ^1^D_NH_ values are found for α-helical segments [22]. Again, a more sophisticated ensemble approach provides information about specific structural properties such as transient secondary and tertiary structures [23,24]. Despite the tremendous success of these applications of RDCs in the past care has to be taken in the case of IDPs and careful control experiments have to be employed to ensure that the conformational ensemble is not significantly perturbed by the anisotropic alignment media. A more comprehensive review of the field is beyond the scope of this perspective article and can be found elsewhere ([25], and references therein).

### 2.4 Paramagnetic relaxation enhancements (PREs)

Undoubtedly the most relevant experimental approach to probe transient long-range contacts in IDPs employs the measurement of paramagnetic relaxation enhancements (PREs) [26]. Since ^1^H–^1^H nuclear Overhauser effects (NOEs) are characterized by pronounced distance dependence conventional NOESY experiments are not sensitive enough to probe distances beyond approximately 6 Å, particularly, as the effective populations of compact sub-states are generally rather small in IDPs. To study paramagnetic relaxation enhancements the protein under investigation is chemically modified by attaching paramagnetic spin labels at defined positions. Typically, the thiol groups of Cys residues (introduced via site-directed mutagenesis) are used to covalently attach the spin label. It has to be noted that the introduction of paramagnetic spin labels into the protein affects both chemical shifts (pseudo contact shifts, PCS) and/or signal intensities via dipolar relaxation between the unpaired electron and the ^1^H^N^ and ^15^N nuclei [27]. Depending on the specific spin label used these effects will be different. Applications of PCS to IDPs are now also feasible due to the availability of novel ligands for lanthanide ions and will be a promising additional tool as PCSs report both on distances and orientations relative to the principal axes frame of the paramagnetic center. So far, however, paramagnetic relaxation enhancement (PREs) was the most common experimental parameter used for the analysis of IDPs’ tertiary structures in solution. The presence of the paramagnetic spin label (e.g. nitroxide moiety, TEMPO or MTSL) leads to an enhancement in the transverse relaxation rates *R*_2_ depending on the inverse sixth power of the distance (1/*r*^6^) between the unpaired electron and the observed nucleus. For the quantitative analysis of PRE data two approaches have been proposed. In the first approach PRE data are converted into distances using well-established methodology [28] that can subsequently be used in, for example, MD simulations to calculate conformational ensembles [29]. A second approach involves a more sophisticated extended model-free model for the time–dependency of PRE effects [25]. Several applications to IDPs have been reported demonstrating the validity of the approach [30–33].

Despite the popularity and the robustness of the PRE approach applications to IDPs are still far from trivial. Firstly, the identification of suitable spin label attachment sites without prior knowledge of the compact structure is not a trivial task as the introduction of the spacious spin label at positions that are relevant for the compact tertiary structure will inevitably perturb the structures. In the worst case, as observed for globular proteins, single point mutations can have detrimental effects on the structural stability of proteins. Thus, additional, entirely primary sequence-based analysis tools are needed for the reliable definition of attachment sites. Secondly, it has been shown that the pronounced distance dependence of PREs can lead to significant bias in the derived ensemble, although this can be partly improved by invoking independent, complementary experimental data (e.g. SAXS) [30].

## 3 New frontiers in IDP research – challenges and future directions

### 3.1 The order–disorder partitioning problem

Recent studies provided some insight into the molecular details of the conformational ensembles populated by IDPs in solution and call for a reassessment of the binary description scheme proposed for proteins lacking a stable tertiary structure [34]. Although proteins differ in terms of tertiary structure stability both ordered and disordered proteins share similar protein folding funnels encoded by the primary sequence leading to distinct residue–residue interaction patterns. The fundamental differences between ordered and disordered proteins are thus merely the heights of energy barriers and the different distributions of thermally accessible conformational substates. As globular proteins can partly populate different unfolded states, conversely in structural ensembles of disordered proteins a significant number of compact structures can also exist stabilized by enthalpically favored long-range interaction patterns similar to stable protein folds. The popular dichotomic partitioning of proteins is thus inappropriate and more sophisticated theoretical concepts based on physico-chemical reasoning have to be involved. The meta-structure was introduced as a novel concept for protein sequence analysis [34]. In this approach a protein is conceived as a network in which individual amino acids represent the nodes whereas edges connecting two nodes indicate spatial proximity in the 3D structure. Of particular relevance is the fact that in this conceptual view the mutual couplings between individual amino acids and the resulting cooperative character of the protein are retained. It has been shown that the network structure of a protein can be calculated exclusively based on primary sequence information and statistical distribution functions derived from the PDB database [34].

**Fig. 3:**
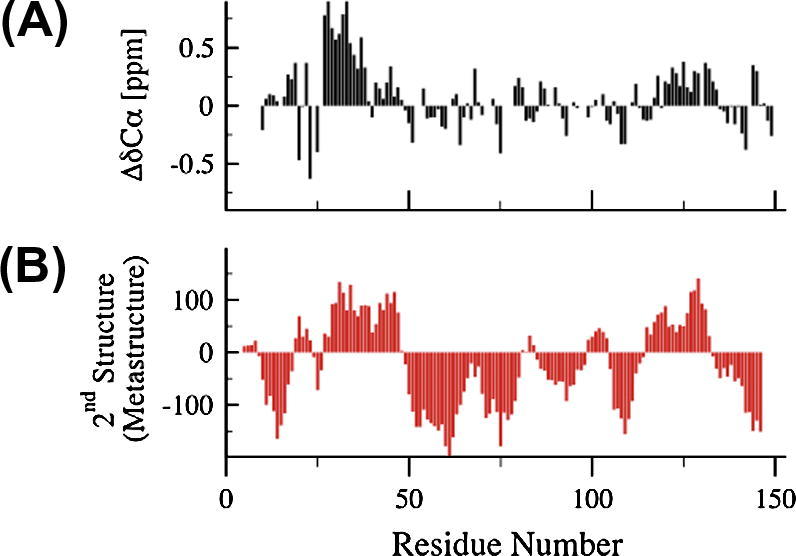
Comparison between experimental NMR data and primary sequence-derived local secondary structure elements in the microtubule binding domain of the IDP MAP1B light chain. (top) ^13^C secondary chemical shifts (ΔδCα); (bottom) meta-structure derived local 2nd structure. The meta-structure derived values are defined according to NMR convention (positive values: α-helical elements; negative: extended conformations or β-strands).

The meta-structure of the protein is quantified by two sequence-derived parameters, compactness and local secondary structure. Residue-specific compactness values quantify the spatial embedding of individual residues within the 3D protein structures. Residues in the interior of a structure exhibit large compactness values while residues located on the surface and exposed to the solvent display small (even negative in case of conformationally highly flexible segments) values. The meta-structure derived secondary structure parameter is defined in analogy to the well-established NMR ^13^Cα chemical shift index, with positive values for α-helices and negative values indicating the presence of an extended conformation. It has already been shown that this novel approach is very useful for the analysis of IDPs [34,35]. Firstly, a large scale comparison of calculated compactness values of IDPs (taken from the DisProt database) with well-folded proteins deposited in the PDB database showed that IDPs display significantly smaller compactness values (∼230) compared to their well-folded counter parts (∼330) suggesting that compactness values are valuable quantitative probes for structural compaction of proteins [34]. Additionally, it was demonstrated that calculated local secondary structure parameters are indicative of α-helices and β-strands [36]. Consistently positive values are found for residues located in α-helical segments while residues populating extended structural elements (β-strands or polyproline II helices) display negative values. A comparison of meta-structure and NMR data for a prototypical IDP is given in Fig. 3. It can be seen that meta-structure values convincingly compare with experimental NMR secondary chemical shifts or NMR-derived secondary structure propensities. Novel applications to large-scale, sequence-based protein analysis and selection (e.g. identification of IDPs displaying significant local α-helical preformation) are feasible and have already been suggested [36].

**Fig. 4:**
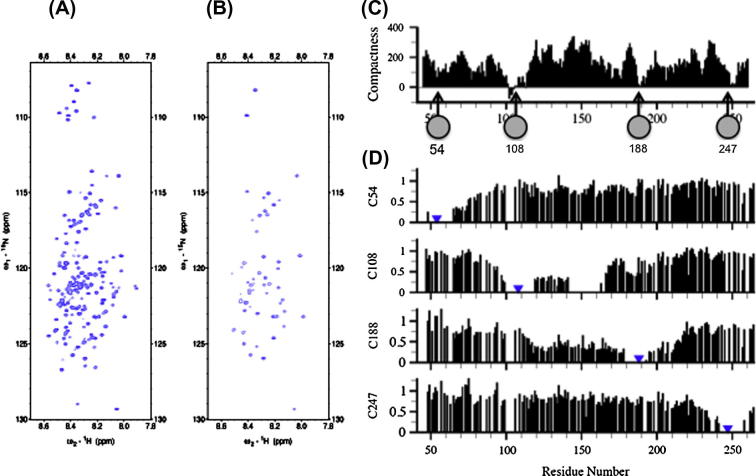
Long-range structural probing in IDPs using paramagnetic relaxation enhancements (PREs). PRE effects are quantified by intensity ratios of cross peaks selected from (A) diagmagnetic reference and (B) paramagnetic (spin-tagged) form of the protein. Selection of suitable spin label attachment sites is based on meta-structure derived compactness values (see text). Large compactness values are found for compact regions of the proteins, whereas small compactness values indicate conformationally flexible residue positions (and thus suitable for attaching the spin label). (C) Meta-structure derived compactness values and (D) experimental PREs obtained on the IDP Osteopontin. Spin label attachment sites were selected using small compactness values [37].

Here another application of meta-structure data (e.g. compactness) is proposed. Despite the ease of experimental implementation, PRE applications are limited due to uncertainties in the identification of suitable spin label attachment sites without prior knowledge of the compact structure. In order to overcome these limitations, it was therefore suggested to use meta-structure derived compactness data to identify suitable sites of spin label attachment [37]. Since residue-specific compactness values quantify the spatial environment of individual residues in 3D protein structures the sites of spin label attachment should therefore be selected based on small compactness values as for these regions tight side chain interactions or packing can safely be neglected. Fig. 4 shows compactness and PRE data for the IDP Osteopontin [37].

### 3.2 Structural adaptations in IDPs

**Fig. 5:**
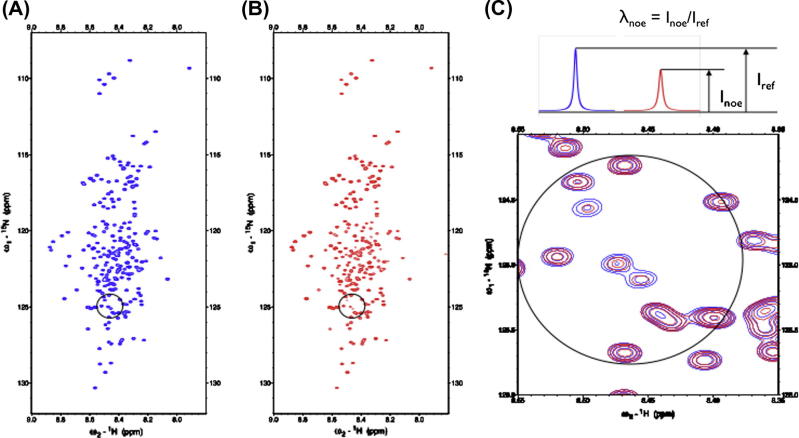
Probing of structural compaction of IDPs using SOFAST-HMQC techniques [38]. SOFAST-HMQC spectra of chicken BASP1 obtained under acidic (pH = 2) conditions without (A) or with (B) inversion of aliphatic side-chain protons. (C) enlarged view (circled) showing differential compaction of the polypeptide chain in BASP1. The intensity ratio *λ*_noe_ between reference (without, *I*_ref_) and by applying the aliphatic proton inversion pulse (*I*_noe_) is indicative of structural compaction of the polypeptide chain.

In addition to their innate conformational flexibility (plasticity) IDPs are also sensitive to changes of environmental parameters (e.g. temperature, pH values, presence of interacting ligands). For example, it was shown that although the thymic hormone Prothymosin-α and α-Synuclein remain natively unfolded under acidic conditions, local secondary structure propensities in proximity to acidic residues change upon variations in pH and the conformational ensemble becomes enriched in compact structures with pronounced local rigidity of the protein backbone. In a recent study, we showed that intrinsically disordered human proteins fold under acidic conditions into more compact structures with higher α-helical content largely due to reduced electrostatic repulsion of negatively charged side chains [36]. This finding suggests that IDP recognition elements can be stabilized by favorable electrostatic interactions across the interaction interface (between proton acceptor located at the surface of the IDP and the acidic proton donor of the interaction partner). In this study NMR spectroscopy was used to verify theoretical predictions [36]. Structural compaction was experimentally verified employing PFG-DOSY experiments together with SOFAST-HMQC techniques (Fig. 5) [38]. SOFAST-HMQC experiments efficiently probe ^1^H–^1^H spin diffusion or NOE effects, when a selective inversion pulse (H^sat^) is applied on aliphatic protons before the start of the pulse sequence. In this experiment, two data sets are recorded with (*I*^sat^) and without (*I*^ref^) the inversion pulse H^sat^. The intensity ratio (*λ*_NOE_ = *I*^sat^/*I*^ref^) depends on spin diffusion effects and quantitatively probes the structural dynamics of proton spin networks [38]. In well-structured, globular proteins spin diffusion is highly efficient leading to *λ*_NOE_ ≪ 1, while in loosely folded proteins (random coils, molten globules) *λ*_NOE_ ≈ 1. In BASP1 (Brain Acid Soluble Protein 1) a significant decrease of *λ*_NOE_ was observed upon lowering pH (0.75–0.60) corroborating the predicted structural compaction of BASP1 under acidic conditions. Given its ease of implementation and reliability of quantitative analysis the SOFAST-HMQC technique will be important for future studies of IDPs’ structural adaptations under varying experimental conditions.

The acid induced compaction of BASP1 was additionally probed by a novel NMR technique that measures ^1^H–^1^H cross-relaxation (NOE) during adiabatic fast passage (AFP). The proposed experiment involves adiabatic fast passage radio-frequency (RF) pulses with a parabolic phase modulation leading to a linear frequency sweep through a considerably large spectral window. In addition to its well-established applications for broadband spin inversion and/or decoupling, the original AFP concept has been used to measure heteronuclear spin lock relaxation rates [39]. In contrast to conventional AFP schemes the RF field intensity is comparable to the frequency sweep range and, thus, leads to increased transverse relaxation contributions to the effective spin lock relaxation rate [39]. If the AFP pulse is applied during a NOESY mixing period a time-dependent weighted combination of NOE and ROE effects is effective. Since NOE and ROE enhancements are of different sign and strength for large molecules, *σ*_eff_ will change sign dependent on the applied radiofrequency field. At weak *ω*_1_ longitudinal cross-relaxation (NOE) dominates the effective spin-lock cross-relaxation rate, while at strong *ω*_1_ transverse cross-relaxation (ROE) prevails and, thus, leads to the characteristic zero crossing of the spin-lock cross-relaxation rate for large molecules, where NOE and ROE cross-relaxation rates cancel. For a rigid macromolecule zero crossing occurs at an effective tilt angle of *θ*_eff_ = 35.26°. Enhanced internal mobility leads to zero crossing at smaller tilt-angles, while spin diffusion effects (for example, in cases where ligands are embedded in hydrophobic pockets) lead to zero passages at larger tilt angles.

**Fig. 6:**
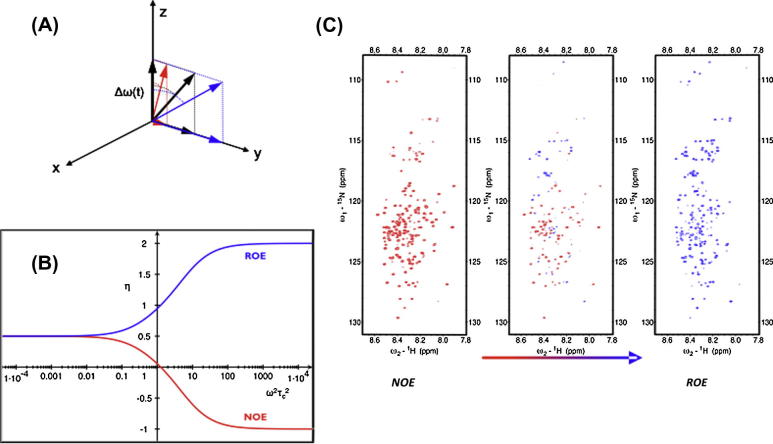
Adiabatic fast passage NOESY as a valuable tool for studies of structural dynamics of IDPs. (A) Adiabatic spin-lock frame with offset Δ*ω*(*t*), and different effective fields *ω*_eff_(*t*). Increasing the RF field strength *ω*_1_(*t*) (from red to blue) leads to an increase of the tilt angle *θ*(*t*) between the static magnetic *B*_0_ (along *z*-axis) and the effective field. Depending on the field strength cross-relaxation during adiabatic fast passage is predominantly longitudinal (red, NOESY-type) for low field strength and dominated by transverse contributions (blue, ROESY-type) at high field strengths. Depending on the effective correlation time (B) AFP-NOESY cross peaks can change sign. (C) ^13^C–^1^H filtered ^1^H^N^–^15^N detected cross-relaxation in the IDP BASP1. Increasing the AFP field strength (from left to right) leads to increased contributions of ROESY-type cross-relaxation pathways. It should be noted that individual residues in the IDP BASP1 display significantly different side-chain backbone cross-relaxation behavior indicating differential mobilities along the protein backbone.

The new experiment for structural probing of IDPs is basically a 3D NOESY-^1^H–^15^N-HSQC experiment with the exception that the AFP pulse replaces the NOESY mixing time and that the initial element recording ^1^H chemical shift evolution is replaced by a ^13^C-filter element to restrict NOE/ROE measurements to the dipole-interaction between aliphatic, ^13^C-attached protons and amide protons. In contrast to a conventional INEPT element, the delay *τ_A_* is chosen so that 2*τ_A_* = 1/*J*_CH_ and, thus, leads to a selective inversion of protons bound to ^13^C-labeled carbons. Experiments are performed twice, with and without *J*_CH_ scalar coupling evolution (^1^H inversion). Signals stemming from ^13^C-bound protons are selected by proper combination of sub-spectra. All other contributions, amide proton to amide proton as well as solvent to amide protons are thus largely suppressed. The results are given in Fig. 6 and demonstrate that the AFP-NOESY experiment is able to probe differential structural compaction of individual backbone positions via ^1^H–^1^H cross-relaxation dynamics. Increasing the AFP spin lock strength (Fig. 6, left to right) clearly changes the cross-relaxation behavior and leads to a shift from NOESY-type to ROESY-type performance. For a protein devoid of internal mobility a passage through zero occurs at the tilt angle of *θ* = 35.3°, while internal mobility and/or spin diffusion effects can alter the tilt angle profile of rotating-frame cross-relaxation rates. The different dependencies observed for BASP1 (Fig. 6) convincingly illustrate the potential of the methodology to probe differential side-chain dynamics in IDPs. In future applications it is planned to extend the methodology to higher frequency dimensions exploiting non-uniform sampling techniques. Details of the sequence and results will be reported elsewhere (manuscript in preparation).

### 3.3 Ligand binding – rational drug design

IDPs are involved in fundamental biological (physiological) processes and are, therefore, of great interest to medical and pharmaceutical research [40]. Their inherent structural flexibility allows them to accommodate different binding partners exploiting different binding modes (e.g. folding-upon binding or formation of fuzzy complexes). Despite limitations due to their unfolded nature several successful studies have been reported demonstrating that IDPs are indeed amenable to drug development programs [41]. However, the dynamic nature of IDPs impairs the application of conventional structure-based drug design strategies. The lack of 3D structures as bottleneck in the pharmaceutical industry is widely recognized and was recently addressed by a combination of information-rich NMR and new protein sequence analysis tools (e.g. meta-structure) [34,42]. It was already demonstrated that the meta-structure analysis provides valid starting points for ligand development by revealing information about the construction of suitable fragment libraries and ligand binding modes [42]. Given the fact that only primary sequence information is needed, valuable applications also to ligand identification for IDPs can be anticipated. A prototypical application to IDPs is given with the example of Osteopontin (OPN), an extracellular matrix protein associated with metastasis of several kinds of cancer. The meta-structure analysis revealed a similarity to the (folded) protein antithrombin. The naturally occurring, highly sulfated glycosaminoglycan heparin is an established ligand for antithrombin. Heparin binding to OPN was verified using NMR spectroscopy [37]. It was shown that heparin binding to OPN causes significant and specific chemical shift changes. This example illustrates how the combined usage of meta-structure and NMR data can be used to create valid starting points for drug development programs involving IDPs. In subsequent steps NMR spectroscopy can be used to provide additional information about binding modes and orientations of bound ligands [42].

**Fig. 7:**
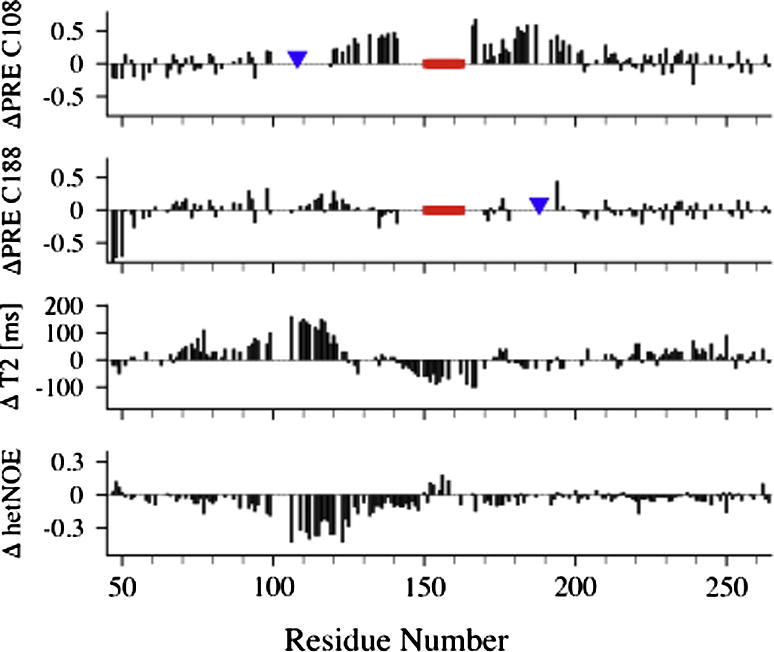
Structural and dynamical adaptations in the IDP OPN upon heparin binding are probed by differential PREs and ^15^N relaxation parameters. (top) Structural changes are probed by differential ΔPRE. ΔPRE > 0 indicates “on average” increasing distance between labeling site and a residue upon binding (for ΔPRE < 0 the opposite). The red bar indicates the heparin binding site (binding site residues were excluded from the analysis because of signal overlap). The location of spin label attachment sites is indicated by blue triangles. (bottom) Conformational dynamics were monitored via ^15^N T_2_ and ^1^H^N^–^15^N NOEs.

Naturally, a comprehensive analysis has to address both structural and dynamical changes. In a recent NMR analysis we have employed both PRE and ^15^N NMR relaxation data to analyze the interaction between the IDP Osteopontin (OPN) and heparin (manuscript in preparation). Fig. 7 shows differential PREs and ^15^N relaxation parameters (^15^N-T_2_ and ^1^H^N^–^15^N NOE). As can be seen OPN exhibits distinct changes of long-range contacts upon heparin binding. In particular, spin labels at C108 and C188 experience displacements from the core region (90–100, 120–150 and additionally from residues 150–200 for C108). Changes of motional dynamics were probed by ^15^N relaxation parameters. The regions containing most of the heparin binding site (140–170 and 180–200) display a decrease in ^15^N T_2_ due to local rigidification of the backbone upon binding (ns time scale motions), paralleled by larger ^1^H^N^–^15^N heteronuclear NOE values indicating reduced fast, picosecond timescale backbone motions. In contrast, the region encompassing residues 90–120 exhibits increased backbone flexibility (increased T_2_ values and more negative heteronuclear NOEs). Interestingly, isothermal titration calorimetry (ITC) measurements provided evidence for significant enthalpy entropy compensation. More details will be given elsewhere (manuscript in preparation). Summing up, it was found that upon binding to heparin, OPN largely retains its disorder and undergoes compensatory (structural and dynamical) adaptations largely mediated through electrostatic interactions. These results indicate the relevance of dynamical adaptations in IDP complexes for thermodynamic compensations and the control of rapid substrate binding and release events in IDP interaction networks.

## 4 Novel experimental tools for IDP research

### 4.1 Local structure probing using cross-correlated relaxation (CCR)

Although NMR chemical shifts are very powerful to probe local structures in proteins additional NMR parameters are desirable to define dihedral angle distributions along the polypeptide chain of IDPs. Cross-correlated NMR relaxation (CCR) has attracted substantial interest in the past as a powerful tool to study structural dynamics of proteins in solution [43]. CCR results from correlated fluctuations of relaxation relevant interaction tensors. In proteins dipole–dipole, dipole–CSA and CSA–CSA cross-correlations are most relevant and several experimental schemes have been proposed. While these experiments have been shown to provide valuable information for globular, folded proteins, applications to IDPs are still limited. As a first example of an application to an IDP, we have recently demonstrated that intra-residue ^1^H(*i*)–^15^N(*i*)–^13^C′(*i*) dipolar–CSA interference can be efficiently used to discriminate between type-I and type-II β-turns in IDPs [44]. The experiment is based on a relaxation pathway originally designed for measurements in globular proteins and was combined with non-uniform sampling techniques required to overcome the spectral overlap problem encountered in IDPs. Since IDPs populate Ramachandran space in a rather unique way and substantially sample β-turn (I,II) and polyproline II helical conformations, this novel experimental approach can be efficiently used to assess these (non α-helical, non β-strand) conformations in IDPs. In this first application the experiment was also used to probe subtle local structural changes in IDPs upon pH-induced structural compaction [44].

**Fig. 8:**
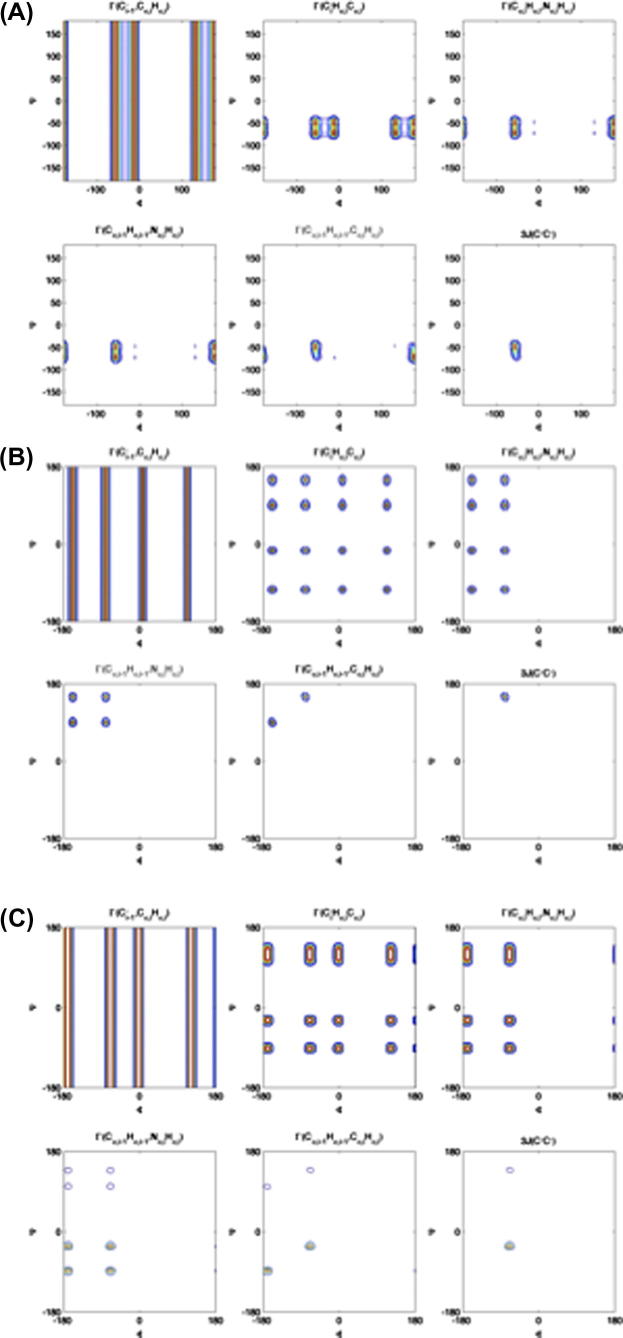
Automated backbone dihedral angle determination using cross-correlated relaxation (CCR). The figure shows a schematic description how the combined usage of different, complementary cross-correlation rates can be used to unambiguously identify the backbone dihedral angles *φ* and *ψ*. Details can be found elsewhere [45]. For illustration purposes different backbone dihedral angle arrangements are shown: (A) α-helical; (B) β-strand. Conformational averaging – as expected for an IDP – is illustrated with an equal distribution between α-helical and β-strand, shown in (C).

It has to be noted, however, that an experimental single cross-correlation rate does not provide unambiguous geometrical information but is consistent with several dihedral angles. To overcome this limitation a method has been suggested exploiting the simultaneous analysis of different complementary cross-correlation rates for the extraction of unambiguous and reliable dihedral angles along the protein backbone [45]. Fig. 8 illustrates the performance of the approach in case of dihedral angle distributions. It can be seen that even in the presence of conformational averaging (e.g. exchange between, for example, α-helix and β-strand) the existence of individual secondary structure elements can be identified. Moreover, it is anticipated that cross-correlated relaxation experiments will be very valuable to complement information about conformational averaging in IDPs stemming exclusively from chemical shift data. While chemical shift data report only on individual spins, cross-correlated relaxation probes coupling between different relaxation mechanisms located at different positions distributed along the protein backbone.

### 4.2 A combined NMR/EPR approach for IDP research

**Fig. 9:**
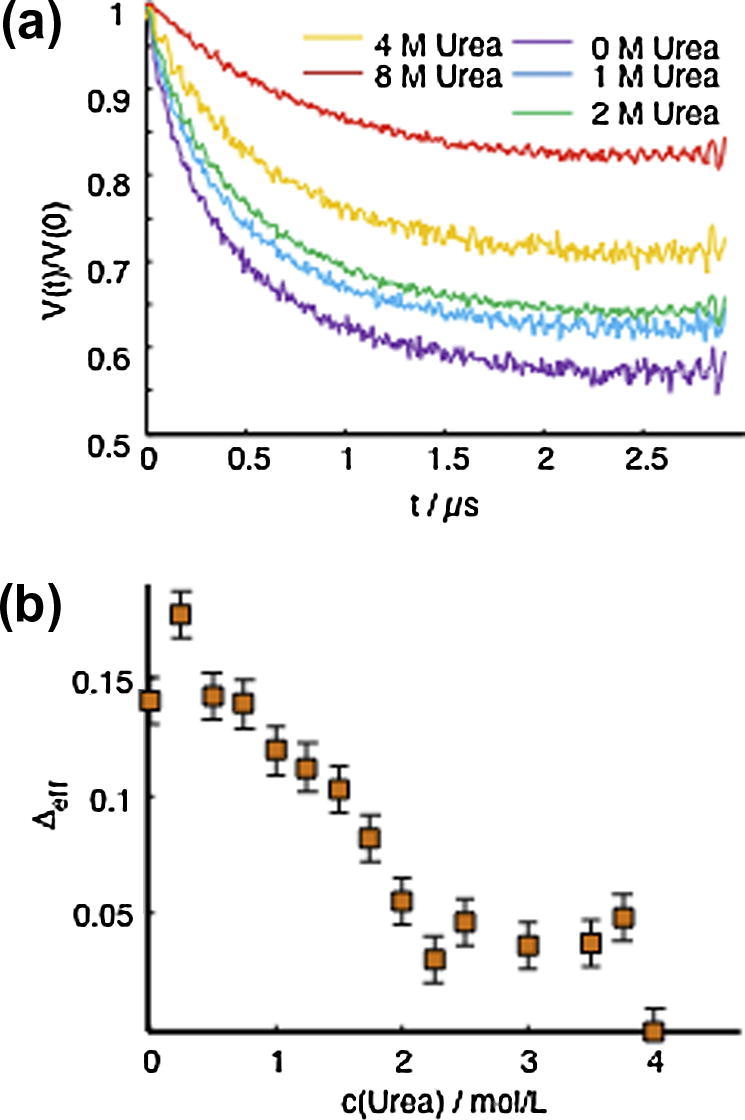
Solution structural probing of IDPs using EPR-based double electron–electron resonance (DEER) spectroscopy [46]. (A) DEER time traces of the double Cys-mutant C108–C188 of the IDP Osteopontin (OPN) at different urea concentrations. The modulation depth, *Δ*_eff_ = 1.0 − *V*(*t* = 3 μs)/*V*(0) is a direct measure of structural compaction [46]. Decreased *Δ*_eff_ at higher urea concentration is due to global unfolding of the protein. (B) Identification of cooperatively folded substates in the ensemble of the OPN by measuring *Δ*_eff_ for the OPN double Cys-mutant C54–C247 as a function of urea concentration compaction. The sigmoidal dependence of *Δ*_eff_ vs urea concentration clearly shows the existence of a cooperatively folded substate [46]. Error bars stem from signal noise.

In addition to the above mentioned, well-established NMR parameters, a novel approach to look at IDPs was recently proposed [46]. It was demonstrated that electron paramagnetic resonance (EPR) spectroscopy offers unique insight into the structural dynamics of IDPs under native conditions as it provides information about the existence of structurally heterogeneous sub-states. While solution NMR provides ensemble averages, pulsed EPR spectroscopy is performed at low temperature where transitions between different states are quenched and individual states can be probed. The methodology was applied to the IDP Osteopontin (OPN), a cytokine involved in metastasis of several kinds of cancer. Structural preferences of OPN were probed by applying the EPR-based method double electron–electron resonance (DEER) spectroscopy to six spin-labeled Cys-double mutants of OPN (C54–C108, C108–C188, C188–C247, C54–C188, C108–C247 and C54-C247). It is important to note that DEER experiments yield non-averaged data and display intramolecular dipole–dipole coupling between the two spins of the labels of a double mutant where the detected signal modulation is related to the dipolar coupling frequency that in turn depends on the interspin distance as *r*^−3^. However, the established analysis tools fail in the case of IDPs as a consequence of the rather broad pair-distribution functions between the two spin labels of a double mutant. Therefore the observed non-modulated DEER data were analyzed through an effective modulation depth, *Δ*_eff_, that is an approximate measure of the average interspin distance for broad P(R)s. *Δ*_eff_ values were measured as a function of urea concentration (Fig. 9). Most importantly, while most of the mutants showed a smooth decrease upon urea denaturation, for the double mutant C54–C247 an unexpected sigmoidal *Δ*_eff_-derived denaturation profile with urea concentration was observed (Fig. 9B). This unexpected and unprecedented sigmoidal urea dependence clearly indicates the existence of stably and cooperatively folded tertiary structures in OPN’s structural ensemble. The conformational ensemble of OPN thus contains both, cooperatively folded and unfolded, extended conformations. Additionally, EPR and NMR (PRE) experiments under high NaCl concentrations showed that not only hydrophobic interactions contribute to the OPN’s structural stability, but also electrostatics play a crucial role in the stabilization of compact structures of OPN in solution [46]. The surprisingly detailed picture of the conformational ensemble of OPN obtained by this novel approach indicates valuable applications to studies of structural dynamics of IDPs.

### 4.3 Molecular details and time scales of conformational sampling in IDPs

**Fig. 10:**
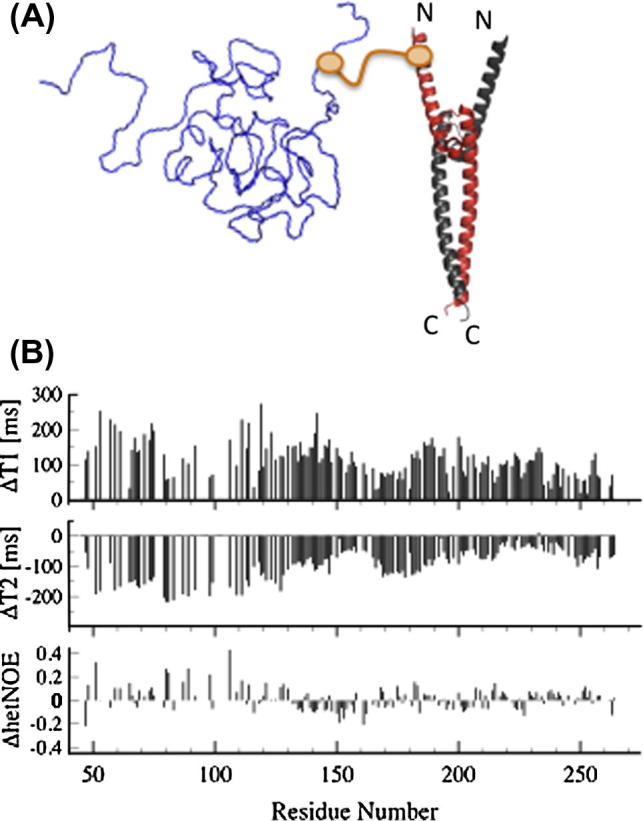
Domain elongation strategy for dynamic studies of IDPs. The presence of collective dynamic modes in IDPs does not allow for the separation of overall and internal dynamics. Significant separation of time scales can be achieved by (covalently) coupling the IDP to a large internal reference frame (fusion protein). (A) The IDP Osteopontin (OPN) (blue) is covalently linked (via Bismaleimide) to the C-terminal interaction domain of the oncogenic transcription factor Myc (C34). Binding of Myc (red) to its cognate protein binding partner Max (gray) leads to the formation of a stable α-helical protein complex displaying significant motional anisotropy (PDB_ID: 1NKP). (B) Differential changes (OPN_Myc-Max_ − OPN_Wild-Type_) of ^15^N relaxation parameters (T_1_,T_2_, ^1^H–^15^N NOE) upon domain elongation (C54@OPN was chosen as the Myc attachment site). The heterogenous distribution of relaxation parameters clearly indicates that OPN does not exist as a random coil in solution but populates compact substates [37,46]. The covalent Bismaleimide linker is indicated in orange.

IDPs are characterized by rugged energy landscapes devoid of distinct energy barriers and therefore display significant structural plasticity and undergo large structural rearrangements. A comprehensive characterization of the solution structures of IDPs thus requires studies of conformational dynamics. NMR spectroscopy is destined for these studies and a plethora of different experiments are available providing detailed information about motional dynamics on different time scales. Fast (ps–ns) time scale motions are probed by ^15^N spin relaxation experiments (^15^N-T_1_,T_2_ and ^15^N–^1^H^N^ NOEs) and analyzed using well-established theoretical frameworks (e.g. model-free formalism [47]). Slower motions occurring on μs–ms time scales are investigated by CPMG-type schemes introduced decades ago and turned into a powerful experimental methodology applicable even to very large molecular weight systems by Kay and co-workers [48]. The particular uniqueness of NMR spin relaxation measurements is the fact that detailed information about internal motions can be discerned. In case of globular, stably folded proteins the analysis relies on distinctly different correlation times describing overall tumbling and internal motions. In case of IDPs this clear-cut separation is no longer valid and thus hampers the application of this approach. A similar situation was encountered in RNA NMR studies. In order to overcome this limitation the group of Al-Hashimi developed an elegant domain elongation strategy to effectively decouple internal motions from overall tumbling [49]. In a similar way we adapted this strategy to relaxation studies of IDPs. As a first example we studied the internal dynamics of OPN using dimeric Myc/Max protein complex for domain elongation. The crystal structure of Myc/Max revealed a four helical bundle structure with significant overall anisotropy. OPN was covalently attached to Myc via a Bismaleimide linker (C54@OPN–C34@Myc). ^15^N NMR relaxation data obtained for the OPN-Myc/Max complex were compared with data obtained from isolated OPN (Fig. 10). It can be seen that the observed distinct changes of relaxation rates as a function of residue position indicate that OPN does not exist as a globally disordered protein in solution but populates compact conformations. Experiments to explore the potential of the methodology to probe conformational dynamics in IDPs (e.g. determination of time scales) are currently underway in the laboratory.

### 4.4 Defining the conformational ensemble

Despite their annotation as unstructured/disordered there is growing NMR experimental evidence that IDPs sample heterogeneous conformational spaces comprising both extended marginally stable conformations as well as stably, eventually even cooperatively folded compact states with distinct arrangements of side-chains [46]. The fundamental problem in the structural characterization of IDPs is thus the definition of a representative conformational ensemble sampled by the polypeptide chain in solution. To date two conceptual approaches are applicable to ensemble calculations of IDPs. The first relies on ensemble averaging using restrained MD simulations or Monte Carlo sampling incorporating experimental constraints as driving force. The second concept assigns populations to a large pool of structures that have been pregenerated by invoking native and/or non-native bias using experimental constraints (e.g. PREs, chemical shifts, RDCs, SAXS) [15–17,23,18]. Given that sampling of the enormously large conformational space accessible to IDPs cannot be exhaustive the question remains how representative the obtained ensembles are.

**Fig. 11:**
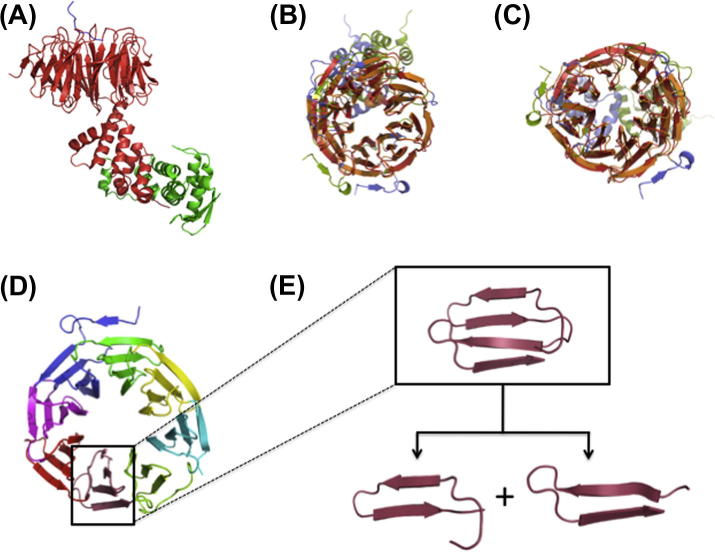
The modular architecture of proteins. (A) Crystal structure of the *Saccharomyces Cerevisiae* Skp1-Cdc4-pSic1 peptide complex (PDB-ID: 3V7D); red: Cdc4:263-744; green: Skp1; blue: pSic1:67-85. The 3D structure of Cdc4 comprises eight repeats of a four β-strand motif which itself results from a duplication of a basic β-hairpin structure. (B) and (C) internal structural superpositions indicating the repeating structure (B: rotation angle = 360/8 = 45°; C: rotation angle = 5 * 360/8 = 225. (D) 8-fold structural symmetry in Cdc4 (the different motifs are color coded). (E) The basic four β-strand structural motif is composed of two fundamental β-hairpins. Internal structural symmetries were identified using the program TopMatch [58].

In this context it is important to note that a similar conclusion was made for the unfolded state of proteins [50]. Experimental findings and theoretical considerations have provided evidence that the unfolded state is not a featureless structural ensemble but rather described as an ensemble of distinct conformations retaining a surprisingly high degree of structural preformation. The enormous reduction of conformational space reconciles the Levinthal paradox [50]. Structural preformation is a consequence of the existence of autonomously folded structural domains which themselves can be decomposed into smaller elements (e.g. super-secondary structure elements, closed loops) [51–53,50]. Detailed analysis of protein structures revealed that the fundamental building blocks of proteins typically consist of residue stretches of 20–25 amino acids length [52]. As an example, Fig. 11 shows a structural superposition analysis and the decomposition of a given protein structure into smaller building blocks with an unexpected high degree of symmetry. A recent bioinformatics study revealed that protein structures can be regarded as tessellations of basic units [54]. This suggests a building principle relying on the existence of pre-defined basic structural motifs that are combined in a combinatorial and – most importantly – (pseudo)-repetitive fashion. A surprisingly simple explanation for this stunning observation of limited protein folds was given by representing the polypeptide chain as a chain of disks or equivalently as a tube of non-vanishing thickness [55]. It was shown that the inherent anisotropy of the chain molecule presculpts the conformational space and thus significantly limits the number of possible protein folds. The inherent symmetry or higher order correlations in protein structures are also relevant in the context of energy landscape theory, as it was predicted that funnelled landscapes and low energy structures are easier to be realized when symmetry prevails [56]. Since IDPs are not at all fundamentally different (based on basic physical chemistry) to their folded counterparts similar principles will be valid in their case, too. It is thus suggested to exploit the fundamental building principles of protein structures for the generation of reliable and meaningful structural ensembles of IDPs by finding and using adequate sequence alignment techniques to identify structural homologues and existing basic motifs. The proposed strategy will rely on a pre-generated large pool of structures from which most suitable conformations are selected using experimental (e.g. PRE, chemical shifts, RDC, SAXS) constraints. Preliminary experiments suggest that meta-structure sequence alignments to sequences taken from the PDB structural database can indeed provide valuable information about hidden similarities and reveal structural building blocks in IDPs that can be subsequently used to improve the quality of the obtained conformational ensembles.

## 5 Conclusion

IDPs display significant structural plasticity and undergo large structural rearrangements of the time-averaged conformational ensemble. Therefore, they seriously challenge classical structural biology that, historically, emphasized only structural aspects of proteins, the spatial arrangements of atoms in proteins and their mutual interactions resulting from a unique conformation. Proteins are characterized by a funnel-like energy landscape and thus do not exist in single conformations but exchange between many different conformational isomers (substates). Fundamental processes or interaction events such as crystallization, protein domain exchanges (swapping), conformational adaptations (e.g. induced-fit) and broad-range binding can be explained as conformational switches (e.g. conformational selection) resulting from the ruggedness of the energy landscape. Most importantly, this conceptual view provides a unified physico-chemical description for both globular and IDPs. While stably folded, globular proteins display a smooth bottom with only few (structurally similar) minima, IDPs have very rugged energy surfaces with low barriers and a large number of accessible minima. The problem of characterizing IDPs has a parallel in the history of polymer science where the introduction of quantitative statistical mechanics models allowed for the successful explanation of the dependence of physical properties of polymeric materials on molecular weight distributions [57]. For the characterization of the properties of IDPs, too, it seems that statistics are required to fully grasp their physico-chemical properties that endow them with unique functionalities. For a comprehensive description of intrinsically disordered proteins and their functionalities clearly information about (1) structure, (2) dynamics and (3) thermodynamics is needed. As outlined in the manuscript, NMR spectroscopy is ideally suited to accomplish these tasks. Well-established methodology already exists that can be used to probe both (1) structure and (2) dynamics of IDPs, but what about (3) thermodynamics? The examples presented in the manuscript indicate that NMR (in conjunction with EPR) can provide valuable information about cooperative effects in IDPs. An important question, however, remains: How do IDPs populate numerous states in their conformational ensemble and what is the relationship between the geometry of the energy landscape and the nature of conformational transitions between different states? In stably folded proteins transitions between different conformational states often occur as (reversible and discontinuous) first-order phase transitions. For IDPs more complex phase transitions can be expected and conformational averaging might also proceed in a continuous manner where the interconverting states coexist and, thus, suggest another level of functional control based on the nature of sampling of the accessible structural space. NMR has already been developed into a uniquely powerful technique to study conformational exchange processes (folding-unfolding processes, phase transitions) and has provided unprecedented insight into the structures and dynamics of low-populated (excited) protein states in solution. Although new computational tools and theoretical concepts will still be needed to properly address the phase behavior of proteins, NMR spectroscopy is undoubtedly destined to play a significant role in this new area of research.
